# Therapeutic Potential of the Nitrite-Generated NO Pathway in Vascular Dysfunction

**DOI:** 10.3389/fimmu.2013.00174

**Published:** 2013-07-02

**Authors:** Michael Madigan, Brian Zuckerbraun

**Affiliations:** ^1^University of Pittsburgh, Pittsburgh, PA, USA

**Keywords:** nitrate, nitrite, nitric oxide, pulmonary hypertension, neointimal hyperplasia, peripheral vascular disease, atherosclerosis, review

## Abstract

Nitric oxide (NO) generated through L-arginine metabolism by endothelial nitric oxide synthase (eNOS) is an important regulator of the vessel wall. Dysregulation of this system has been implicated in various pathological vascular conditions, including atherosclerosis, angiogenesis, arteriogenesis, neointimal hyperplasia, and pulmonary hypertension. The pathophysiology involves a decreased bioavailability of NO within the vessel wall by competitive utilization of L-arginine by arginase and “eNOS uncoupling.” Generation of NO through reduction of nitrate and nitrite represents an alternative pathway that may be utilized to increase the bioavailability of NO within the vessel wall. We review the therapeutic potential of the nitrate/nitrite/NO pathway in vascular dysfunction.

## Introduction

The Nobel Prize in physiology or medicine was awarded to Drs. Furchgott, Ignarro, and Murad in 1998 for their work in identifying nitric oxide (NO), previously recognized as endothelium-derived relaxing factor, as a biologic mediator of the cardiovascular system. Since that time, NO has been extensively researched and has been linked to numerous physiological and pathological processes within the cardiovascular system. Vascular dysfunction is the root cause of a variety of important disease processes, including myocardial infarction, stroke, peripheral vascular disease, pulmonary hypertension, and wound healing. This constellation of pathology imposes a significant financial burden on the healthcare system and produces significant morbidity and mortality in those affected. The underlying pathophysiology of vascular dysfunction occurs in numerous forms, and often involves a combination of dysregulated endothelial cell NO production, increased proliferation and migration of smooth muscle cells, increased formation of intimal and medial plaques, impaired collateral vessel generation, and reduced angiogenesis.

## The l-Arginine/Nitric Oxide Pathway

**Figure 1:**
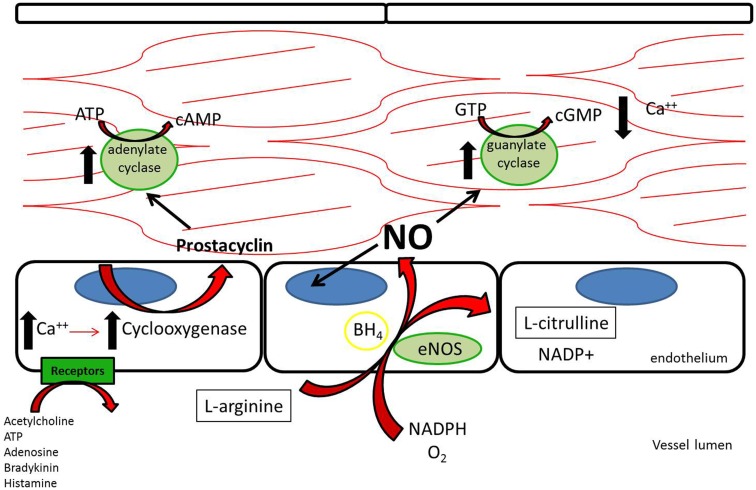
**L-Arginine is metabolized in endothelial cells via endothelial nitric oxide synthase to nitric oxide, which then acts downstream to reduce platelet adhesion, decrease leukocyte adhesion, inhibit smooth muscle proliferation and migration, and induce vasodilation**. Acetylcholine, adenosine triphosphate, adenosine, bradykinin, and histamine all act on different receptors to generate downstream prostacyclin, which acts as a redundant system to induce vasodilation and platelet inhibition (112).

Three nitric oxide synthases (NOSs), nNOS (neuronal), iNOS (inducible), and eNOS (endothelial), were identified and initially thought to be the sole producers of NO within the cardiovascular system (1). Both nNOS and eNOS are calcium-dependent and constitutively active, while iNOS is induced under inflammatory conditions and is calcium-independent. All three isoforms metabolize l-arginine, NADPH, and oxygen to l-citrulline, NADP, and NO (2) (Figure 1). l-arginine may alternatively be metabolized by arginase to l-ornithine and urea. When the supply of l-arginine is limited, metabolism via arginase may effectively reduce production of NO (3).

**Figure 2:**
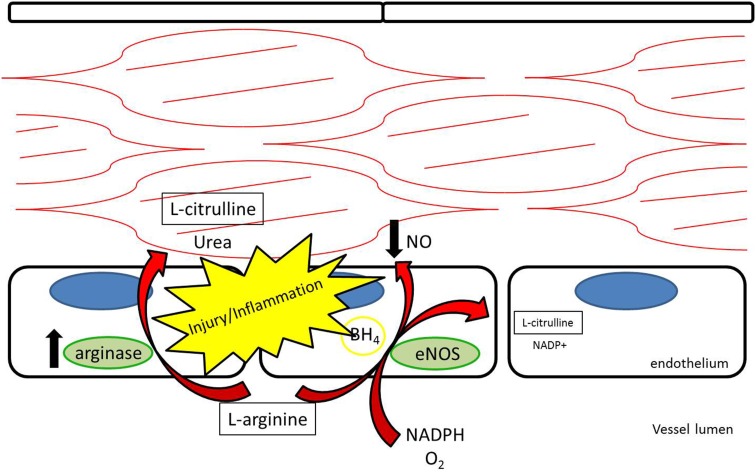
**L-arginine may be competitively metabolized by arginase to L-citrulline and urea, reducing production of nitric oxide and contributing to vascular dysfunction**.

It has been suggested that the shunting of l-arginine away from the NOS/NO pathway toward the arginase/l-ornithine pathway contributes to certain vascular pathology (4–7) (Figure 2). Expression of arginase in the vascular wall is induced under pro-inflammatory conditions, as well as by reactive oxygen species (ROS) and reactive nitrogen species (RNS) (8). Increased arginase activity has been associated with hypertension and coronary vascular dysfunction (9–11). Also, direct vascular injury induces a local inflammatory response. Arginase is upregulated in the vessel wall after balloon injury in the rat carotid injury model. Polyamines generated through the l-ornithine pathway form the building blocks necessary for smooth muscle cell proliferation and neointimal hyperplasia of the vessel wall (12). Peyton et al. (13) demonstrated that selective inhibitors for arginase attenuate neointimal hyperplasia in the rat carotid injury model.

**Figure 3:**
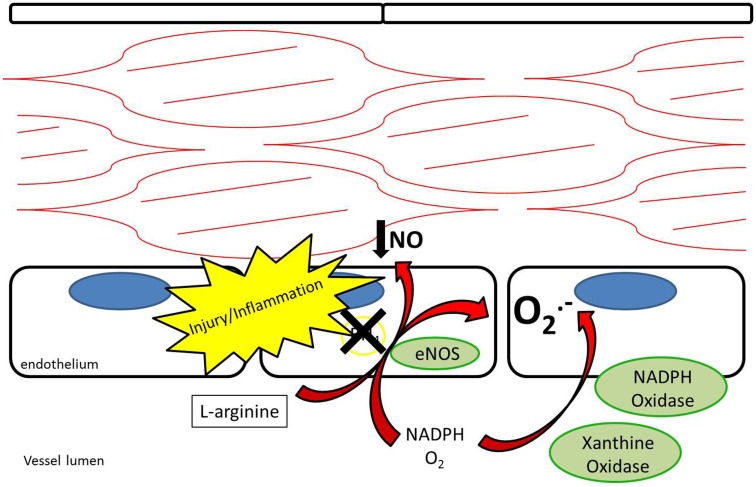
**Endothelial nitric oxide synthase uncoupling results in reduced production of nitric oxide as well as production of superoxide anions**. NADPH oxidase and xanthine oxidase also contribute to production of superoxide anions.

Endothelial NOS is highly expressed in endothelial cells at baseline. Its metabolism of l-arginine to NO is thought to be a major contributor to plasma nitrite levels, which play an important role in baseline vasodilation (14, 15). In addition to regulating baseline vasomotor tone, eNOS is thought to help limit platelet adhesion and thrombosis (16, 17). After vessel injury iNOS is upregulated in arterial smooth muscle cells and eNOS is upregulated in the endothelium resulting in increased NO production (18). Under pathological conditions, the increased NOS activity may not translate into increased NO production. Reduced NO bioavailability through eNOS “uncoupling” is a contributing factor to reduced local NO in atherosclerosis, pulmonary hypertension, and vessel injury (7, 19). Tetrahydrobiopterin (BH_4_) is an essential cofactor for the enzymatic production of NO via NOSs (20). Uncoupling occurs under conditions of reduced BH_4_ availability where eNOS produces superoxide anions rather than NO (21, 22) (Figure 3). In addition, ROS are produced by NADPH oxidase and XOR (23, 24). ROS have been recognized as contributing to vascular dysfunction, through mechanisms including endothelial dysfunction, vascular smooth muscle cell growth, lipid peroxidation, and inflammation (25). An alternative source of NO under these conditions may help restore the NO deficiency attributed to uncoupling.

## Nitrate/Nitrite Reduction to Nitric Oxide

**Figure 4:**
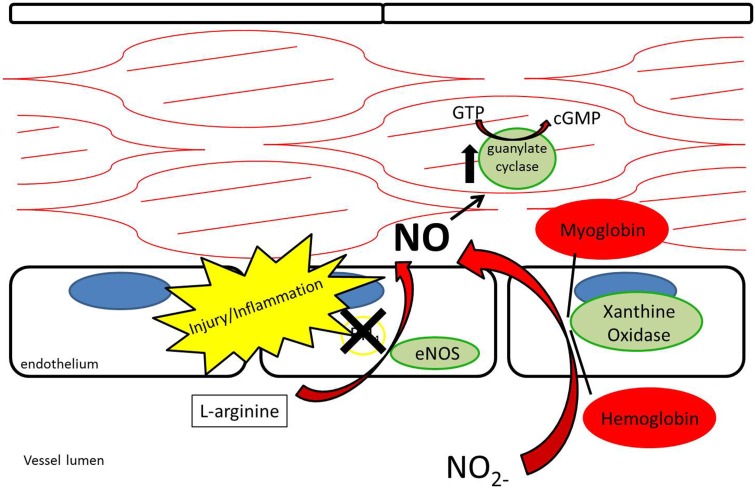
**Nitrite reduction by xanthine oxidoreductase, myoglobin, hemoglobin, and protonation results in nitric oxide production, especially under conditions of hypoxia and acidemia**.

While nitrate and nitrite were long thought of as stable end-products of NO metabolism, recent evidence supports nitrate and nitrite as potential sources of NO under appropriate conditions (12, 26–29) (Figure 4). As opposed to the NOS enzymes, which require oxygen as a substrate for NO generation, nitrite-generated production of NO has been shown to occur more readily under acidic and hypoxic conditions (30–32, 113). Nitrate/nitrite reduction has been shown to occur via deoxygenated hemoglobin, myoglobin, enzymatic, and non-enzymatic means (33–37). A class of molybdenum-containing enzymes, including xanthine oxidoreductase (XOR), aldehyde oxidase (AOX), and sulfite oxidase (SUOX), have been identified as enzymes that may facilitate the reduction of nitrate and nitrite to NO at the molybdenum-containing site (38). We and others have shown that XOR in particular is present within the vessel wall and tissue and contributes to NO production in intimal hyperplasia, pulmonary hypertension, and ischemia-reperfusion (12, 26, 39).

While l-arginine is a significant contributor to plasma nitrite production through the l-arginine/NOS/NO/nitrite pathway, plasma nitrite levels are also dependent on oral consumption of nitrate and nitrite (40). The Mediterranean diet, which has been associated with a lower risk of atherosclerosis and coronary artery disease, adds credence to the importance of oral nitrate/nitrite-derived NO in vascular biology (41, 42). The Mediterranean diet, known for its high content of nitrate-rich leafy green vegetables, has also been found to lower the blood pressure of healthy volunteers (40, 43). The nitrate/nitrite/NO pathway through oral ingestion is thought to rely on a symbiotic relationship with natural oral flora. Nitrate is concentrated within the salivary glands and salivary bacteria reduce nitrate to nitrite in the oral cavity (44). Once nitrite reaches the stomach, it is reduced to NO by protonation due to the stomach’s low pH (45). NO then may act locally by enhancing mucosal blood flow to the stomach (45–47). Nitrite is also absorbed in the stomach where it enters the blood stream (48). Due to its relative stability, nitrite then has the ability to circulate to other areas in the body and undergo reduction to NO under acidic and hypoxic conditions (33). Acting in this way, circulating nitrite has been described as a “storage pool” for NO within the body (27).

Historically, there has been concern that oral nitrate/nitrite consumption may increase the risk of some cancers, including esophageal, stomach, and colon cancer. Some epidemiological studies have suggested that high oral intake of nitrate/nitrite correlates with increased risk of gastrointestinal malignancy, though accuracy in calculating dietary exposure is difficult (49). Nitrosylation of secondary amines via nitrite occurs readily under acidic conditions, such as in the stomach, resulting in N-nitrosamines. Around 300 N-nitrosamines have been identified as carcinogenic (50). The National Toxicology Program (51) of the US Department of Health and Human Services found no evidence of carcinogenic activity in mice and rats after 2 years of exposure to oral sodium nitrite. The International Agency for Research on Cancer (52), a division of the World Health Organization, evaluated the evidence concerning dietary consumption of nitrate/nitrite and carcinogenicity in their monographs. The IARC concluded that inadequate evidence exists in humans and experimental animals for the carcinogenicity of nitrate in food and drinking water and limited evidence exists to suggest carcinogenicity of nitrite in food and drinking water (52). The IARC did, however, recognize that sufficient evidence exists in experimental animals to suggest the carcinogenicity of nitrite in combination with amines or amides and that nitrite in food is correlated with stomach cancer (52). Ongoing research will help elucidate the specific conditions in which *N*-nitrosamines may be carcinogenic in humans.

Multiple investigations have demonstrated that the nitrate/nitrite/NO pathway has vasoactive properties in the systemic and pulmonary circulations. Infusion of nitrite into the forearm brachial artery increased local blood flow and decreased blood pressure at rest and during exercise in humans (33). The infusion correlated with an increase in erythrocyte iron-nitrosylated hemoglobin, suggesting that hemoglobin may play a role in transporting NO through the bloodstream. Dietary supplementation has the potential to achieve similar results systemically. Larsen et al. (43) used a 3-day dietary supplementation of nitrate (0.1 mmol/kg body weight) in healthy volunteers and showed an increase in plasma nitrate (178 ± 51 vs. 26 ± 11 μM) and nitrite (219 ± 105 vs. 138 ± 38 μM). After 3 days, the volunteers also had a decrease in diastolic and mean blood pressure by 3.7 mmHg and 3.2 mmHg, respectively. In a similar study using a Japanese diet high in nitrate, Sobko et al. (53) demonstrated an increase in both salivary and plasma levels of nitrate and nitrite. These volunteers had an average 4.5 mmHg drop in diastolic blood pressure after 10 days. Dietary nitrate may also effect the pulmonary circulation. In mice exposed to hypoxia to induce pulmonary hypertension, dietary nitrate reduced vascular remodeling and right ventricular hypertrophy through pulmonary vasodilation (26). Inhaled nitrite is an alternative delivery method that has the potential to induce pulmonary vasodilation while minimizing systemic effects. Nebulized sodium nitrite reduced hypoxia-induced pulmonary hypertension in lambs by 65% with no drop in systemic blood pressure (54).

## Nitric Oxide and the Vessel Response to Injury

Nitric oxide has been shown to serve many vasoprotective properties that occur after vessel injury, including reduction of platelet deposition, decrease in leukocyte adhesion, inhibition of smooth muscle cell proliferation and migration, and induction of vasodilation (55). One of the initial responses to endothelial disruption is platelet activation and plug formation. NO and NOS expression are associated with decreased platelet adhesion at the vessel wall (56, 57). NO has been shown to be a potent inhibitor of platelet adhesion, reducing thrombosis within the vessel lumen (58, 59). NO mediates platelet adhesion though upregulation of platelet-soluble guanylate cyclase production of cyclic GMP. Nitrate and nitrite-supplemented diets increase bleeding times in mice, and there is an inverse relationship between blood nitrate/nitrite levels and platelet function (60). After platelet deposition, neutrophils and macrophages begin to infiltrate the vessel wall. NO inhibits leukocyte adhesion and the subsequent vessel inflammatory response after injury (61, 62). Once the inflammatory response sets in, smooth muscle cells infiltrate the medial layer and begin proliferating. The resulting thickened medial layer narrows the lumen and stiffens the vessel wall. NO acts to reduce the smooth muscle cell response in multiple ways. NO was first recognized as the substance responsible for calcium-dependent relaxation of the vascular smooth muscle cells (63). NO upregulates soluble guanylyl cyclase within cells and leads to increased cyclic GMP. Cyclic GMP then interacts with protein kinases to lower cytoplasmic calcium, which results in vasodilation (64). Also, it has been shown in culture that NO reversibly arrests the cell cycle of vascular smooth muscle cells (65). NO inhibits smooth muscle proliferation within the vessel wall via a p21 dependent mechanism (66–68). Overall, NO reduces smooth muscle cell migration and proliferation, which can lead to atherosclerosis and neointimal hyperplasia (69).

## Nitric Oxide and Atherosclerosis

Atherosclerosis resulting in coronary artery disease and stroke are the leading causes of death in the developed world (70). Atherosclerotic plaques are formed when the endothelial layer is damaged and cholesterol accumulates within the vessel wall. Macrophages are recruited to the site of injury, form foam cells, and release cytokines leading to an inflammatory response (71). Smooth muscle cells then migrate and proliferate within the vessel wall, eventually leading to an organized plaque (72). Repeated vessel wall injury causes thrombosis and narrowing of the lumen, which leads to ischemia of the tissue bed supplied by the vessels.

While atherosclerosis is a multifactorial process, dysregulation of the arginine/NOS balance contributes to the development of atherosclerotic disease (73). For instance, iNOS inhibition in the apolipoprotein E knockout mouse model for atherosclerosis accelerates the progression of atherosclerotic disease in these mice (74). Restoring the balance of NO production at multiple points along the pathway reduces formation of atherosclerotic plaques. l-Arginine supplementation has been shown to improve vasodilation in cholesterol-fed rabbits and monkeys and reduce the progression of atherosclerosis (75–77). Also, exogenous expression of iNOS in the arteries reduces the injury response and atherosclerotic development (78). Furthermore, supplemental oral nitrite has also been shown to be beneficial in reducing vessel inflammation and endothelial dysfunction in mice treated with a high cholesterol diet (79).

NOS enzyme dysregulation results not only in reduced NO availability, but also increased superoxide anions and arginase activity, both of which are detrimental to maintaining healthy vasculature (80, 81). Oxidized low-density lipoproteins (OxLDL) caused by the interaction between LDL and superoxide anions correlate with atherosclerotic disease (82). OxLDL is taken up by macrophages, which forms foam cells on the vessel wall (73). OxLDLs also have been shown to induce apoptosis of endothelial cells and impair endothelium-dependent arterial relaxation within atherosclerotic vessels (83–85). On the contrary, NO has been shown to inhibit apoptosis in endothelial progenitor cells caused by oxidized low-density lipid proteins (86).

## Nitric Oxide and Peripheral Arterial Disease

Nitric oxide is an important regulator of the tissue response to peripheral arterial disease and lower extremity ischemia, specifically enhancing arteriogenesis, angiogenesis, and progenitor cell migration (4, 87, 88). Arteriogenesis is a recognized phenomenon that involves the enlargement of pre-existing collaterals as a result of increased sheer stress, often in response to stenotic or occluded primary vessels. Angiogenesis, on the other hand, is induced by vascular endothelial growth factor and occurs in response to tissue ischemia (89). As a result, new capillaries are formed (90). Endothelial NOS knockout mice show impaired arteriogenesis, angiogenesis, and pericyte recruitment after femoral artery ligation. All three processes are reversed in this model by intramuscular injection of adenovirus encoding eNOS, suggesting that NO is an important mediator of these processes during lower extremity ischemia (91).

In addition to eNOS-generated NO, the nitrite/NO pathway is functional in the peripheral vasculature. Intraperitoneal (IP) nitrite injections have been shown to improve tissue perfusion through increased collateral vessel development in the murine femoral ligation model of acute limb ischemia (92). IP delivered nitrite also improved angiogenesis and cutaneous flow in rat ischemic myocutaneous flaps, reducing tissue death via a nitrite/NO pathway (93). Nitrite therapy, delivered even in a delayed fashion, augments arteriogenesis in the mouse hindlimb ischemia model (94). Additionally, dietary nitrate supplementation increased capillary and bone-marrow derived progenitor cell density in ischemic hind-limbs, a process that was inhibited with antiseptic mouthwash (95). Antiseptic mouthwash reduces the concentration of the oral bacteria responsible for nitrate reduction to nitrite, thus disrupting the nitrate-nitrite-NO pathway (96). In a small study of healthy volunteers, antiseptic mouthwash increased systolic and diastolic blood pressure by 2–3.5 mm Hg during a 7 day course (97).

## Nitric Oxide and Neointimal Hyperplasia

Neointimal hyperplasia is an exaggerated inflammatory healing response after vascular injury. Of particular interest is neointimal hyperplasia after balloon angioplasty and vascular stent deployment, since this may limit therapeutic success. After vessel injury, platelets adhere to the vessel wall denuded of endothelium and generate a cascade of events leading to leukocyte chemotaxis, extracellular matrix modification, endothelial cell apoptosis, and vascular smooth muscle cell migration and proliferation (55). NO has been shown to limit neointimal hyperplasia through multiple levels. Similar to atherosclerosis, NO modulates neointimal hyperplasia through inhibition of platelet aggregation, decreased leukocyte chemotaxis, and reduced vascular smooth muscle cell proliferation while stimulating that of endothelial cells (57–59, 62, 65, 67, 68, 98, 99). The effects of NO may be limited by l-arginine shunting away from eNOS to arginase under pathological conditions. Arginase metabolism of l-arginine leads to the production of polyamines utilized in cell proliferation, and the expression of arginase I is increased in the proliferation of rat aortic smooth muscle cells (100). It has been demonstrated that arginase I activity is increased within the vessel wall after carotid balloon injury in rats, and that inhibition of arginase decreases neointimal hyperplasia in that model (13). Furthermore, Alef et al. (5) demonstrated that nitrite-supplemented drinking water acts to reduce intimal hyperplasia in the rat carotid injury model, and that this NO is generated through XOR.

## Nitric Oxide and Pulmonary Arterial Hypertension

Pulmonary hypertension is a vascular disease characterized by hypoxia, pulmonary vasoconstriction, increased vascular resistance, vessel remodeling, thrombosis, and right ventricular strain (7, 101). Multiple etiologies likely contribute to the development of pulmonary hypertension, but all involve increased vascular resistance as a prominent factor. NO, an important regulator of pulmonary vascular resistance, acts as a vasorelaxing agent within the pulmonary arterial system as well as a protective agent against smooth muscle cell proliferation within the vascular wall (102, 103). It has been proposed that NO may act as a “hypoxic buffer” that leads to vasodilation under hypoxic conditions, such as occurs in pulmonary hypertension (104, 105). This theory proposes that increased nitrite reduction to NO helps to counterbalance the hypoxic pulmonary vasoconstriction by generating a vasodilatory signal. Inhaled nitrite is being utilized in pulmonary hypertension as a direct means of delivering NO to the pulmonary vasculature (106). Also, dietary nitrite in mice increases pulmonary dilation, inhibits vascular remodeling, and decreases right ventricular hypertrophy. This effect was reduced in eNOS knockout mice and after allopurinol treatment (26). In a rat model of pulmonary hypertension, it has been shown that inhaled nitrite reverses the effect of hypoxia-induced pulmonary hypertension through creation of NO via XOR (103).

Investigation into the l-arginine/nitrite/NO pathway in pulmonary hypertension has led to conflicting results as far as the importance of this system. Variation in eNOS expression has been observed in human tissue studies, despite consistently elevated eNOS in animal studies (107–109). Inducible NOS has also been shown to be increased in some studies (110). The upregulation of the NOSs may be a compensatory response to upregulated arginase activity. Like other vascular disorders, arginase activity has been shown to be increased in pulmonary hypertension (111). Increased arginase may have a dual role of decreasing l-arginine metabolism to NO as well as polyamine-induced increases in smooth muscle cell proliferation within the vessel walls (7).

## Summary

Nitric oxide is an important regulator of vascular function. An imbalance in NO production in relation to ROSs, RNSs, and other inflammatory mediators is associated with many forms of vascular dysfunction, including atherosclerosis, peripheral arterial disease, neointimal hyperplasia, and pulmonary hypertension. The recently discovered nitrate/nitrite/NO pathway is an alternative means of delivering NO to areas of deficiency. In order to harness this pathway as a therapeutic, efficient delivery to the affected tissues must be accomplished. Because of its relatively stable nature and the recognition that nitrate, nitrite, hemoglobin, and myoglobin within the blood act as a ‘storage pool’ of NO, a variety of potential delivery options to areas of vascular dysfunction exist, including dietary supplementation, inhalation, and direct intravenous infusion.

## Conflict of Interest Statement

The authors declare that the research was conducted in the absence of any commercial or financial relationships that could be construed as a potential conflict of interest.
